# Chronic Stress-Induced Gene Changes *In Vitro* and *In Vivo*: Potential Biomarkers Associated With Depression and Cancer Based on circRNA- and lncRNA-Associated ceRNA Networks

**DOI:** 10.3389/fonc.2021.744251

**Published:** 2021-09-28

**Authors:** Ting Zhou, Mingming Li, Zhijun Xiao, Jian Cai, Weiwei Zhao, Jingjing Duan, Zhen Yang, Zhijun Guo, Yitian Chen, Weijia Cai, Piaopiao Huang, Chaoyong He, Feng Xu

**Affiliations:** ^1^ Department of Pharmacology, China Pharmaceutical University, Nanjing, China; ^2^ Department of Pharmacy, Fengxian Hospital, Southern Medical University, Shanghai, China; ^3^ Department of Pharmacy, The First Affiliated Hospital of Guangzhou Medical University, Guangzhou, China; ^4^ Department of Pharmacy, Fengxian Mental Health Center, Shanghai, China; ^5^ Department of Laboratory Medicine, Fengxian Hospital, Southern Medical University, Shanghai, China; ^6^ Department of Central Laboratory, Fengxian Hospital, Southern Medical University, Shanghai, China; ^7^ Department of Pharmacy, The International Peace Maternity & Child Health Hospital of China Welfare Institute, Shanghai, China

**Keywords:** ceRNA network, circRNA, lncRNA, depression, cancer, microarray analysis

## Abstract

Circular RNAs (circRNAs) and long noncoding RNAs (lncRNAs) have been considered as biomarkers or regulators in many diseases. However, the exact role of circRNA- or lncRNA-mediated competing endogenous RNA (ceRNA) networks in the modulation of depression pathogenesis-relevant processes is not clear. In this study, we profiled whole transcriptome in depression patients’ blood samples *via* microarray analysis. As a result, a total of 340 circRNAs, 398 lncRNAs, 206 miRNAs, and 92 mRNAs were differentially expressed between the depression and control groups. Then, we constructed ceRNA networks according to the differentially expressed genes (DEGs). Using bioinformatics analysis, 89 pairs of circRNA-ceRNA and 49 pairs of lncRNA-ceRNA networks were obtained. Since depression is a broad and heterogeneous condition that is known as promoter for many chronic diseases including cancer, so we further dug out 28 circRNAs, 61 lncRNAs, 26 miRNAs, and 29 mRNAs that are associated with cancer. Gene Ontology (GO) and Kyoto Encyclopedia of Genes and Genomes (KEGG) analyses showed that the DEGs were significantly enriched in cancer-related signaling pathways such as MAPK, Wnt, IL-17, Ras, and PI3K-Akt. Genes involved in the above pathways such as S100A9, GATA2, SRFP5, SLC45A3, NTRK1, FRZB, has_circ_0014221, has_circ_0014220, and has_circ_0087100 were dysregulated in various cancer cell lines by stress hormones induced. HDC, GATA2, SLC45A3, and NTRK1 were downregulated in tumor-bearing mice subjected to chronic unpredictable mild stress (CUMS). LncRNA-mediated ceRNA network validation showed that overexpression of miR-4530 declined HDC level. Our findings highlight the potential circRNA- and lncRNA-mediated ceRNA regulatory mechanisms in the pathogenesis of depression and as potential biomarkers in depression cancer comorbidity through the pathways of IL-17 or histidine metabolism.

## Introduction

Depression is a broad and heterogeneous condition. About 22.1% of people worldwide suffer from depression and other mental health problems (1). Depression is not always a separate disease, it is often accompanied by other diseases including diabetes and cancer (2, 3). Many studies have shown that depression increases cancer risk, accelerates cancer progression, and aggravates cancer prognosis (4–6). Up to now, the diagnosis of depression is still based on depression rating scale assessment and clinical symptom analysis; no effective biomarkers are available in clinical practice, not to mention the biomarkers which might be useful for predicting depression cancer comorbidity.

Noncoding RNAs (ncRNAs) including microRNAs (miRNAs), lncRNAs, and circRNAs play key roles in the development and progression of many diseases. CircRNAs and lncRNAs regulate gene expression *via* sponging miRNAs (7). Over the past few years, remarkable progress has been made in establishing circRNAs or lncRNAs as important regulators or biomarkers of various biological processes in several diseases (2, 7–10). For example, circ0093740 promotes tumor growth and metastasis by serving as a sponge of miR-136/145 (11). LINC00998 promoted HCC tumorigenesis by encoding a small endogenous peptide (12). However, the expression profiles and possible roles of these ncRNAs and their potential function in depression remain largely unknown.

In the present study, blood samples of six depression patients and six healthy volunteers were collected and analyzed using microarray. Differentially expressed circRNAs, lncRNAs, miRNAs, and mRNAs were selected for deep analysis. We conducted ceRNA networks (circRNA-miRNA-mRNA and lncRNA-miRNA-mRNA) of the DEGs followed by GO and KEGG pathway analyses to elucidate the potential functions of these ceRNAs. Since depression is known as a promoter for many chronic diseases including cancer, we further identified some cancer associated RNAs that were enriched in the IL-17 signaling pathway and histidine metabolism as the important genes, to detect their expression in various cancer cell lines induced by stress hormones as well as in tumor-bearing mice disturbed by CUMS. This work aims to provide novel perspectives for depression/cancer comorbidity-associated ncRNA biomarker research and a ceRNA mechanism of depression aggravating cancer.

## Materials and Methods

### Patients and Clinical Samples

Twenty-seven outpatients diagnosed as mild to major depression and 26 healthy volunteers were recruited for the study. Patients with pregnancy or lactation were excluded. Detailed characteristics and scores of Hamilton Rating Scale for Depression (HAMD24), Life Event Scale (LES), and Negative Life Events score (NLEs) for each patient were conducted. This study was carried out in compliance with the principles of the Declaration of Helsinki. The procedures for this study were approved by the Ethics Committee of Fengxian Hospital, Southern Medical University (KY201608), and all participants provided written informed consent.

### Blood Collection

Peripheral venous blood samples were collected from each participant in PAXgene TM Blood RNA Tubes. The tubes were kept upright at room temperature (25°C) for 6 h, then transferred into a freezer (−20°C) overnight, and finally stored at a temperature of −80°C. The samples were used for total RNA/miRNA extraction. A portion of each blood sample was placed in a coagulation tube and allowed to clot for 2 h at room temperature before centrifugation for 15 min at 1,000×g for plasma collection for ELISA assay.

### Cell Lines and Cell Culture

All cell lines (MCF-7, MDA-MB-231, A549, SW480, CAOV3, U87, and LLC) were purchased from Chinese Academy of Sciences cell bank (shanghai, China) and preserved by our lab. Cells were cultured in Dulbecco’s modified Eagle’s medium (DMEM, Gibco, USA) with 10% FBS (Invitrogen, USA), and 1% penicillin/streptomycin. All cell lines were cultured at 37°C with 5% CO_2_. The miR-4530 mimics and control mimics used in this study were designed and synthesized by Hanbio Biotechnology Co., Ltd. (Shanghai, China). MDA-MB-231, A549 and CAOV3 cells were transfected using RNAifectin (CatG073, abm lnc, USA), harvested after 48 h for subsequent experiments.

### Animal Experiments and Sample Collection

Six weeks old female C57BL/6 mice were purchased from Shanghai JSJ Laboratory Animal Co., Ltd. (Animal Quality Certificate: SCXK (Shanghai) 2021-0006) and maintained in a SPF-grade lab individually in cages for 1 week to adapt to the environment. The mice were randomly divided into two groups (nine mice per group): A: tumor group; B: CUMS+tumor group. Mice in the B group were subjected to chronic unpredictable mild stress (CUMS) for a total duration of 8 weeks as previously described (13) to establish the CUMS model. Then mice in both groups were implanted subcutaneously with one million of LLC cells in 100 µl 1:1 of PBS. Animal procedures were performed according to the guidelines of the Institutional Ethics Committee of Fengxian Hospital, Southern Medical University. When the tumors reached the end point, all mice were sacrificed and xenograft tumors were photographed and weighted, then fixed with formalin and/or snap-frozen.

### Verification of CUMS-Induced Depression-Like Behavior

Open-field test Depression-like behaviors were examined using open-field test (OFT) and sucrose preference test (SPT). The open-field behavior of each mouse in both groups was analyzed in a quiet room for 5 min, using a ZS-ZFT Video Analysis System (ZSDC Science and Technology Co., Ltd., Beijing, China) before and after the CUMS. The mice were placed individually in the middle of an open-field apparatus (100 cm × 100 cm × 40 cm). The open-field area was divided into 20 × 20 cm^2^ equal-size squares. Standing times and crawling square numbers were monitored as an index of exploratory behavior and locomotion activity, respectively.

#### Sucrose Preference Test

The sucrose preference test procedure was performed before and after CUMS as described below. The mice of both groups were adopted in single cage and exposed to 1% sucrose solution (w/v) and water, presented at random positions for 24 h. Two bottles were weighed at 0 and 24 h to calculate the water consumption and sucrose consumption. The sucrose preference was calculated by the equation as follows: Sucrose preference = sucrose consumption (g)/(water consumption (g) + sucrose consumption (g)) × 100%.

### ELISA Measurement of Noradrenalin, Cortisol, 5-HT, IL-6, IFN-γ, and TNF-α in Plasma

To further distinguish between depressed and heathy persons, biochemical indicators noradrenalin (NE) (Cusabio, Houston, TX, USA), cortisol (Cusabio, USA), and 5-HT (Jianglai Biotech, Shanghai, China) were measured by ELISA Kit according to the manufacturer’s instructions. Inflammatory factors interleukin 6 (IL-6), tumor necrosis factor alpha (TNF-α), and interferon-γ (IFN-γ) were also measured (Jianglai Biotech, China).

### Microarray Analysis for circRNA, lncRNA, mRNA, and miRNA

For the whole transcriptome expression profiling, Human circRNA+lncRNA+mRNA Array v3.0 was used for CircRNA, lncRNA, and mRNA microarray analyses (4*180K, Design ID:078298). Agilent human miRNA, Release 21.0 (8*60K, Design ID:070156) microRNA Array was used for miRNA microarray analysis. Chip hybridization was performed according to the hybridization standard process and supporting kit provided by the Agilent expression profiling chip. After washing, the arrays were scanned by Agilent Scanner G2565CA (Agilent Technologies, Inc., Santa Clara, CA, USA). Analysis method for transcriptome RNA data was as follows: data were extracted with Feature Extraction software 10.7 (Agilent technologies, Santa Clara, CA, USA) and normalized by quantile algorithm, limma packages in R (http://www.r-project.org/).

### Gene Ontology and KEGG Pathway Analysis

To further investigate the potential function of the differentially expressed genes (DEGs), the biological pathway and functional classification of these genes were performed using GO terms (http://geneontology.org/) and KEGG pathways (http://genome.jp/kegg/). GO terms and KEGG pathways with *p*-values <0.05 were considered to be significantly enriched.

### ceRNA Network Construction

First, predict miRNA target gene using the miRanda software, miRDB (http://mirdb.org/), and TargetScan (http://www.targetscan.org/vert_72/). Second, Mircode online tool (http://www.mircode.org/) was performed to predict miRNA-circRNA and miRNA-lncRNA. Third, circRNA-mRNA and lncRNA-mRNA regulatory relationship pairs were predicted based on consensus miRNAs. Finally, synthesize the prediction results of ceRNA_score and P value to predict ceRNA triplets. The ceRNA score and P value for the potential ceRNA pairs in this method were calculated using the formulas listed below. (The closer the ceRNA score is to 1 and the smaller the *p*-value is, the more credible the result is): The circRNA-miRNA-mRNA or lncRNA-miRNA-mRNA ceRNA networks were visualized using Cytoscape software (version 3.6.1) (14). In addition, GO and KEGG pathway analyses for the ceRNA networks were performed using the hypergeometric distribution test method.


$$\text{ceRNA}\_\text{score}=\frac{\#\text{MRE}\_\text{for}\_\text{share}\_\text{miRNA}}{\#\text{MRE}\_\text{for}\_\text{circRNA}\_\text{miRNA}}$$

$$p=\sum_{i=mc}^{\min(m_{p},m_{n})}\frac{\left(\begin{array}{c} m_{n}\\ i \end{array}\right)\left(\begin{array}{c} M_{T}-m_{n}\\ m_{p}-i \end{array}\right)}{\left(\begin{array}{c} M_{T}\\ m_{p} \end{array}\right)}$$

where *M_T_* represents the number of all miRNA; *m_p_* the number of miRNA-regulating mRNA; *m_n_* the number of miRNA-regulating circRNA or lncRNA; and *m_c_* the number of shared miRNAs. *p* < 0.05 was considered a potential ceRNA pair.

### RNA Extraction and Quantitative Real-Time PCR Validation

For microarray analysis, ethylenediaminetetraacetic acid (EDTA)-treated whole blood samples of depression patients and healthy volunteers were collected. Total RNA was extracted using TRIzol reagent (Carlsbad, CA, USA) and purified using PAXgene™ Blood RNA Kit (Cat#762174, QIAGEN, GmBH, Hilden, Germany) following the manufacturer’s instructions. Then, a RIN number was checked to inspect RNA integration by an Agilent Bioanalyzer 2100 (Agilent Technologies, Santa Clara, CA, USA). Six samples of RNA in both groups were chosen randomly for microarray analysis while others were left for quantitative real-time PCR (qRT-PCR) validation.

For qRT-PCR validation, total RNA was extracted from whole blood/cancer cell lines/tumors with TRIzol. miRNA was extracted from whole blood for miRNA validation. cDNA was synthesized by using a Reverse Transcription Kit for circRNAs, lncRNAs, mRNAs (Takara, Shiga, Japan), or cDNA Synthesis Kit for miRNA (Genenode Biotech Co., Ltd., Beijing, China, Cat# 4205), followed by RT-qPCR analyses with SYBR Green qPCR Mix (Genenode Biotech Co., Ltd., Cat# 4302). GAPDH (for circRNA, lncRNA, and mRNA) and U6 (for miRNA) were used as internal controls. For miR-4530 and miR-1470, the primers (one RT primer and a pair of qPCR primers) were designed by RiboBio. Stem-loop method was used to synthesize cDNA and determined using Bulge-loop™ miRNA RT-qPCR kit (RiboBio, Guangzhou, China). The sequences of other primers are shown in **Supplementary Table S1**. Relative DEGs were calculated using the 2^−ΔΔCt^ formula. All experiments were replicated three times.

### Statistical Analysis

For microarray data analysis, differentially expressed RNAs were identified through Log|FC| >1 and *p*-value (≤0.05). Volcano plot, heat map, GO and KEGG analyses of the gene expression data were carried out with R packages. Statistical analysis of other data was performed with GraphPad Prism-6; *t*-test (two tailed) was used to assess statistical significance of the qRT-PCR and ELISA results between the two groups. The Pearson correlation coefficient was conducted to analyze the correlations between gene expression and HAMD scores. Data were expressed as mean ± standard deviation (SD). *p < 0.05 was considered statistically significant.

## Results

### Validation of Depression

**Figure 1:**
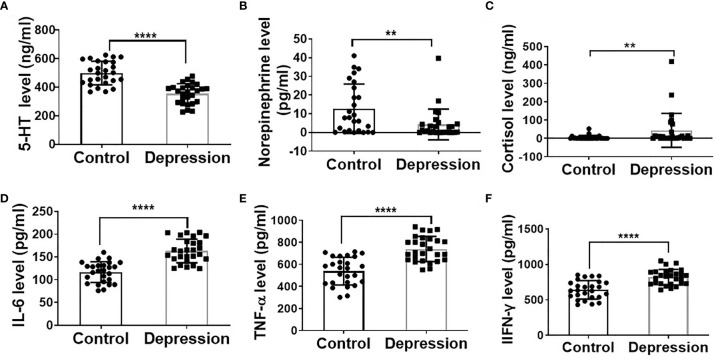
Depression altered hormone levels and elevated inflammatory cytokine levels. Plasma levels of 5-HT **(A)**, norepinephrine **(B)**, cortisol **(C)**, IL-6 **(D)**, TNF-α **(E)**, and IFN-γ **(F)** in depression patients (*n* = 27) and mental healthy volunteers (*n* = 26); **p < 0.01, ****p < 0.0001.

As shown in **Supplementary Table S2**, clinical characteristic results showed that the depression patients had significantly higher HAMD24, LEC, and NLE scores. The decreased plasma levels of 5-HT, noradrenalin, and increased cortisol level in the depression group further demonstrate the depression state of the patients (**Figures 1A–C**). Additionally, plasma inflammatory factor levels of IL-6, IFN-γ, and TNF-α were significantly increased in the depression group compared with the healthy ones (**Figures 1D–F**).

### Dentification of DEGs Between Depression Patients and Healthy Volunteers

**Figure 2:**
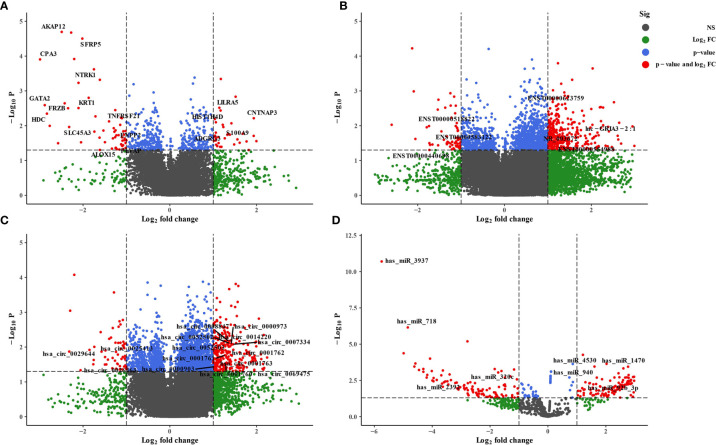
Volcano plot of differentially expressed RNAs between depression patients and health volunteers. |log2(fold change)| >1, false-discovery rate (FDR) <0.05. **(A)** mRNAs, **(B)** lncRNAs, **(C)** circRNAs, and **(D)** miRNAs. Red points with log2(fold change) <−1 represent downregulated RNAs, while red points with log2(fold change) >1 represent upregulated RNAs.

We employed the whole blood of six depression patients and six healthy controls for circRNA, lncRNA, miRNA, and mRNA microarray analyses. Using the unified criteria: Log|FC| >1 and *p* < 0.05, all the DEGs were screened as shown in **Table 1**. Overall, 340 differentially expressed circRNAs were identified, including 285 upregulated and 55 downregulated circRNAs in the depression group compared with the healthy ones (**Figure 2C**; **Supplementary Table S3**). A total of 398 significantly altered lncRNAs (333 were upregulated and 65 were downregulated) were obtained (**Figure 2B**; **Supplementary Table S4**). Using the same stringent criteria, 206 miRNAs were found to be differentially expressed between the two groups, including 125 upregulated and 81 downregulated ones (**Figure 2D**; **Supplementary Table S5**). Ninety-two differentially expressed mRNAs were obtained, among which 34 were upregulated and 58 were downregulated (**Figure 2A**; **Supplementary Table S6**).

**Table 1:** Numbers of differentially expressed genes.

RNAs	Upregulate	Downregulate	Total change
circRNA	285	55	340
LncRNA	333	65	398
miRNA	125	81	206
mRNA	34	58	92

### qRT-PCR Validation

**Figure 3:**
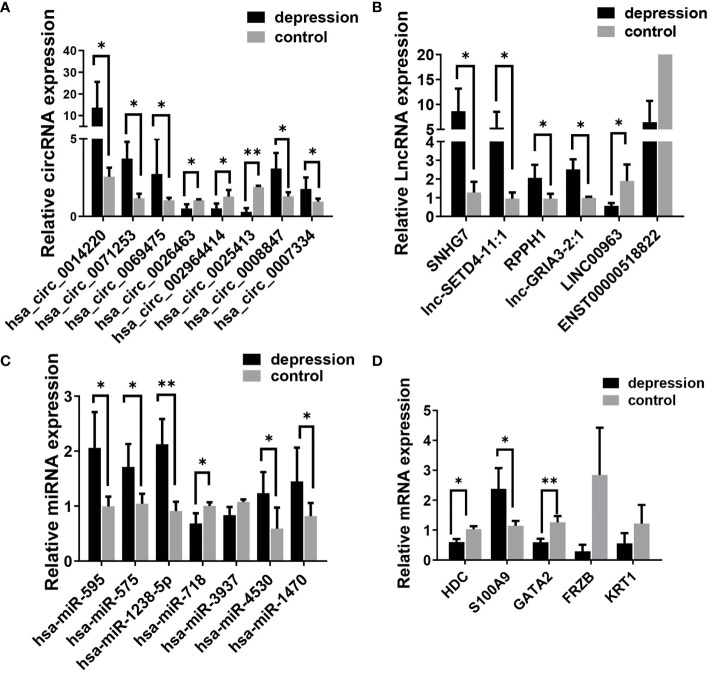
Validation of differentially expressed RNAs selected randomly by quantitative RT-PCR. **(A)** circRNAs, **(B)** lncRNAs, **(C)** miRNAs; and **(D)** mRNAs. Expression was normalized to U6 for miRNAs and GAPDH for others as internal controls. Data are presented as means ± SD (*p < 0.05, **p < 0.01) (*n* = 5).

To verify the results of the microarray analysis, some of the DEGs were randomly selected and further analyzed by qRT‐PCR. As is shown in **Figure 3**, expression levels of all the selected RNAs were consistent with the microarray analysis results, except for has-miR-718, LINC00963, Keratin 1 (KRT1), and frizzled related protein (FRZB); they were supposed to be changed but had no significant differences between the two groups.

### Functional Annotation and Enrichment Analysis of the DEGs

**Figure 4:**
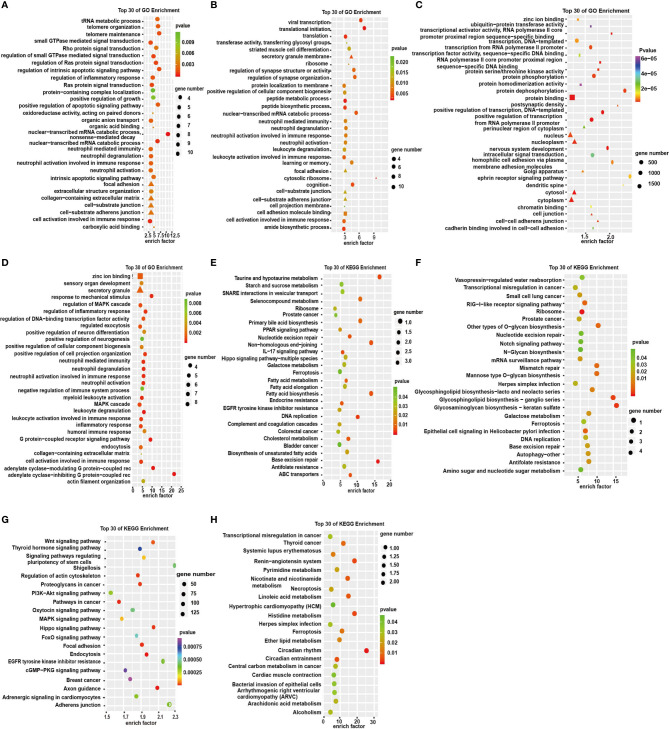
GO enrichment analysis and KEGG pathway analysis in the significantly DEGs. **(A–D)** GO enrichment analysis of top 30 terms of molecular functions (MF: ■), cell components (CC: ▲), and biological processes (BP: ●) of circRNAs, lncRNAs, miRNAs, and mRNAs, respectively. **(E–H)** Top 30 terms of KEGG signaling pathways of circRNAs, lncRNAs, miRNAs, and mRNAs respectively.

To investigate the functions of the dysregulated RNAs, we conducted GO and KEGG enrichment analyses. The GO enrichment analyses showed the GO enrichment of biological processes (BP), cellular components (CC), and specific molecular functions (MF) of differently expressed genes. GO analysis of DE circRNAs indicated that the BP was primarily enriched in the cell activation involved in immune response, small GTPase-mediated signal transduction, and Ras protein signal transduction (**Figure 4A**). KEGG pathway analysis showed that DE circRNAs were mostly enriched in IL-17 signaling pathway, endocrine resistance, prostate cancer, and colorectal cancer (**Figure 4E**). lncRNA pathway enrichment analysis of the DEGs revealed clusters of GO categories and functional pathways such as cellular process, metabolic process, biological regulation, binding and catalytic activity, small cell lung cancer, prostate cancer, transcriptional misregulation in cancer, P53 signaling pathway, and ribosome (**Figures 4B, F**). The results of miRNA showed that the primary molecular function was protein binding; the primary cell components were nucleoplasm, nucleus, cytosol, and cytoplasm (**Figure 4C**). The primary pathways were Wnt signaling pathway, pathways in cancer, and endocytosis (**Figure 4G**). GO analysis of DE mRNAs showed that the BP terms were associated with MAPK cascade, inflammatory response, and leukocyte activation involved in immune response. CC term is enriched in secretory granule and MF term in zinc ion binding (**Figure 4D**). The KEGG pathway analysis was performed to manifest some vital pathways, including transcriptional misregulation in cancer, necroptosis, circadian rhythm, and circadian entrainment (**Figure 4H**). These results suggest that these GO terms and KEGG pathways may contribute to the pathogenesis and biochemical characteristics of depression in humans. Therefore, we presumed that these pathways may be potential therapeutic targets for depression as well as diseases comorbid with depression.

### Construction of the ceRNA Network

**Figure 5:**
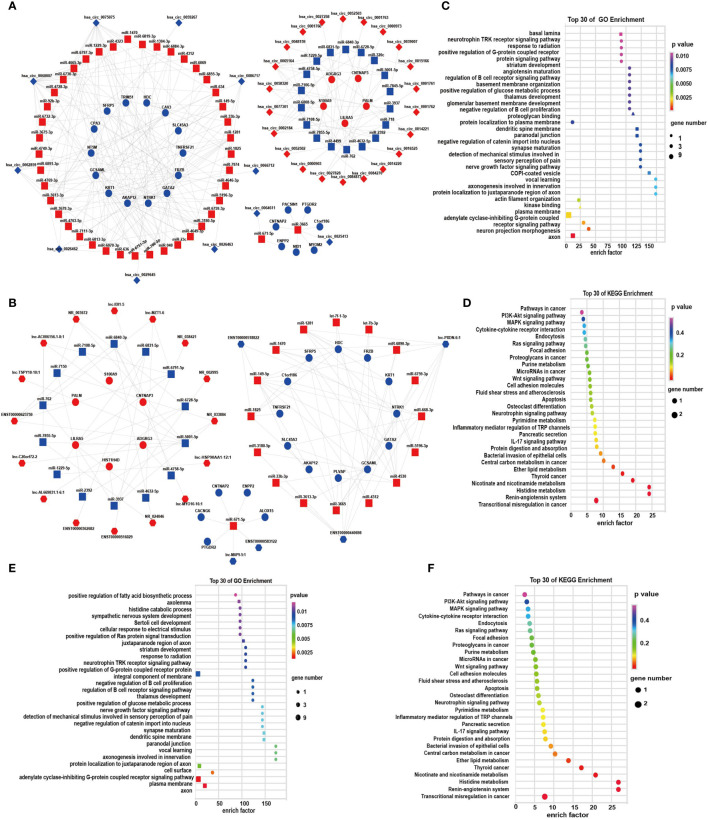
The circRNA/lncRNA-associated ceRNA networks, GO, and KEGG enrichment. **(A)** ceRNA network of circRNA-miRNA-mRNA; **(B)** ceRNA network of lncRNA-miRNA-mRNA, diamond-, hexagon-, square-, and circle-shaped nodes represent circRNA, lncRNA, miRNA, and mRNA, respectively. Red and blue represent upregulation and downregulation, respectively. **(C, D)** GO and KEGG enrichment of circRNA associated ceRNA networks, respectively; **(E, F)** GO and KEGG enrichment of lncRNA-associated ceRNA networks, respectively. (BP: ■; CC: ▲; MF: ● in GO analysis).

For more insights into the roles of DEGs in the progression of depression, according to the ceRNA theory, we constructed global cicRNA-miRNA-mRNA and lncRNA-miRNA-mRNA ceRNA regulatory networks. In the cicRNA-miRNA-mRNA network, 89 pairs of ceRNAs were obtained, which contained 26 mRNAs, 61 miRNAs, and 33 circRNAs (**Figure 5A**; **Supplementary Table S7**). Forty-nine pairs of ceRNA triplets of lncRNA-miRNA-mRNA were also constructed, which contained 23 mRNAs, 31miRNAs, and 21 lncRNAs (**Figure 5B**; **Supplementary Table S8**). These data showed tight correlations and regulatory relationships among the DEGs. In the cirRNA-ceRNA network, has_circ_0000903, has_circ_0001763, has_circ_0052503, has_circ_0001761, has_circ_0052502, has_circ_0001762, has_circ_0001760, and has_circ_0000973 were identified as ceRNAs for miR-4499 to regulate the expression of ADGRG3. In the lncRNA-ceRNA network, PLVAP, SFRP5, SLC45A3, HDC, NTRK1, FRZB, GATA2, and KRT1 may interact with lncRNA Lnc-PXDN-6:1 mediated by miR-4530.

To further investigate ceRNAs of DE RNAs, we then performed GO and KEGG analyses. GO enrichment of circRNA-miRNA-mRNA focused on the adenylate cyclase-inhibiting G-protein-coupled receptor signaling pathway and the neurotrophin TRK receptor signaling pathway (**Figure 5C**). KEGG analysis revealed that the DEGs in the ceRNA networks were associated with the pathways of MAPK signaling, Wnt signaling, IL-17 signaling, Ras signaling pathway, histidine metabolism, microRNAs in cancer, central carbon metabolism in cancer, thyroid cancer, and transcriptional misregulation in cancer (**Figure 5D**). Those composed of the lncRNA-miRNA-mRNA gene were enriched in the integral component of the plasma membrane (**Figure 5E**). Similar results were obtained in KEGG pathway enrichment of lncRNA-ceRNA network (**Figure 5F**).

### Tumor-Associated Gene Screening

**Figure 6:**
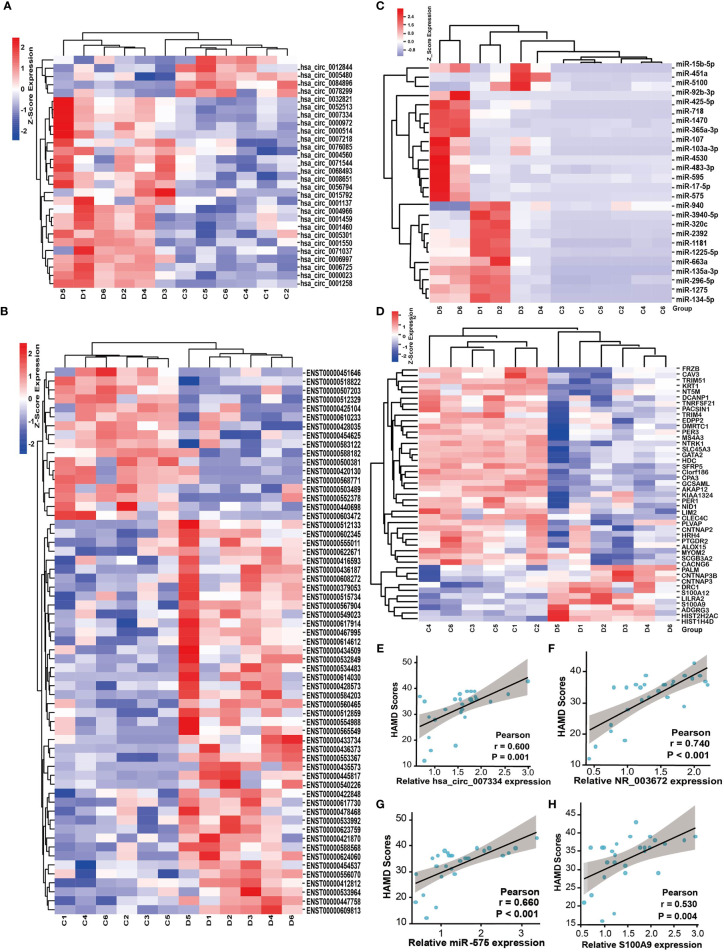
Depression is positively correlated with cancer-associated genes. Heat map of tumor-associated circRNAs **(A)**, lncRNAs **(B)**, miRNAs **(C)**, and mRNAs **(D)** identified from the DEGs, respectively. D, depression patients; C, healthy controls. Red indicates high relative expression and blue indicates low relative expression. FC >2 or FC <0.5, *p* < 0.05. **(E–H)** The correlation between HAMD scores of depression patients and the relative expression of tumor-associated genes circ-0007334 **(E)**, NR_003672 **(F)**, miR-575 **(G)**, and S100A9 **(H)**, respectively.

Depression is known to aggravate cancer progression. It is worth noting that our above results showed that the DEGs were enriched in many tumor-associated pathways. Therefore, we further explored tumor-related genes from the DEGs by screening databases and published literatures (tumor-related circRNA database (15) (https://hanlab.uth.edu/cRic/), Lnc2Catlas Database (16) (https://lnc2catlas.bioinfotech.org/home/), miRCancer, TCGA, etc.). We identified 28 circRNAs, 61 lncRNAs, 26 miRNAs, and 29 mRNAs that are associated with cancer (**Figures 6A–D**). Interestingly, most of the changing trend of the DEGs was consistent with previous reports or the databases. For instance, the oncogene S100A9 was upregulated while the tumor suppressor genes HDC, AKAP12, SFRP5, and ALOX15 were downregulated in depression groups. miR-103a-3p, miR-718, has-miR-3940-5p, etc. were downregulated in miRCancer Database, which were also decreased in the depression group compared with healthy controls. We conducted correlation analysis between the HAMD score and some of the tumor-related DEGs. All the upregulated genes such as circ-0007334, NR_003672, miR-575, and S100A9 were positively correlated with the HAMD scores (**Figures 6E–H**). This may indicate that the severity of depression is positively correlated with cancer.

### Stress-Induced Cancer-Associated Gene Abnormal Expression *In Vitro* and *In Vivo*

**Figure 7:**
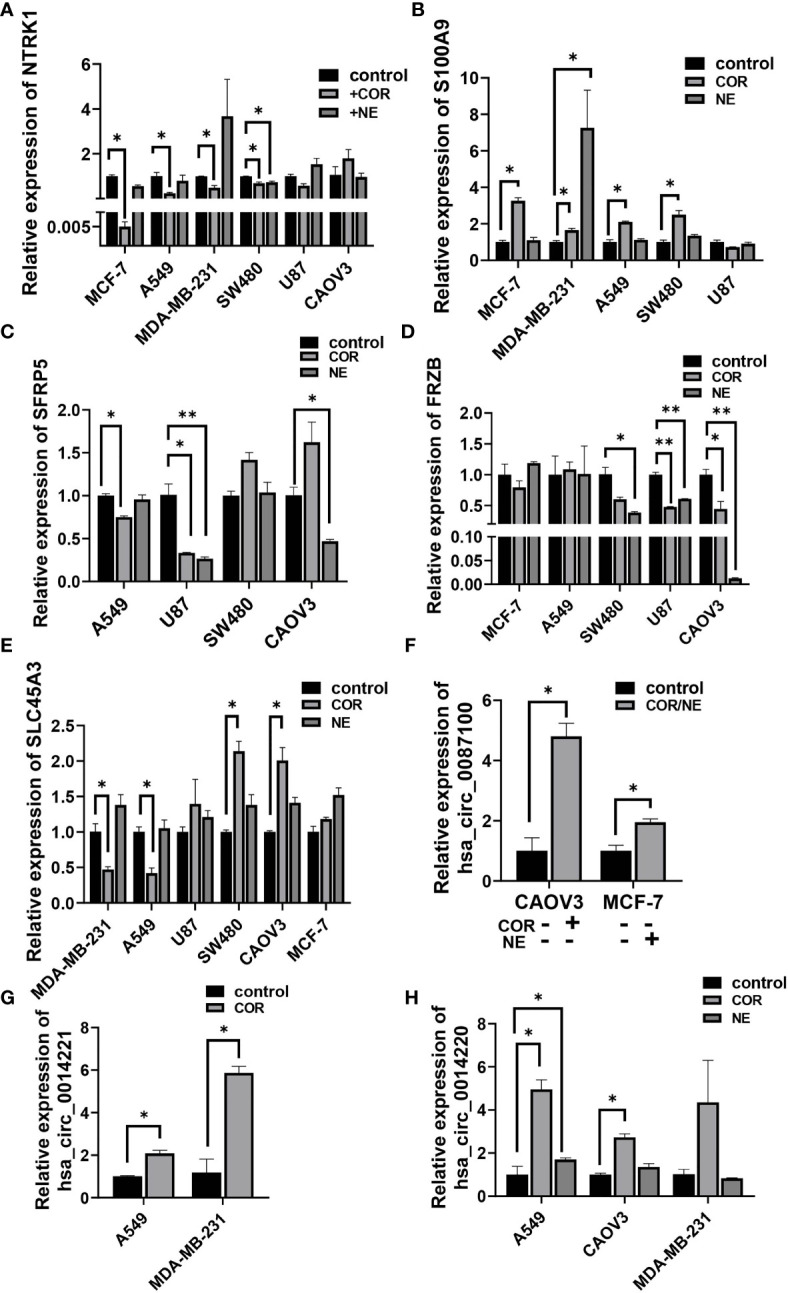
Stress hormones alter the expression of cancer-related genes in various cancer cell lines. qRT-PCR analysis of the relative expression of NTRK1 **(A)**, S100A9 **(B)**, SFRP5 **(C)**, FRZB **(D)**, SLC45A3 **(E)**, hsa_circ_0087100 **(F)**, hsa_circ_0014221 **(G)**, and hsa_circ_0014220 **(H)** in A549, MCF-7, SW480, U87, CAOV3, and MDA-MB-231 cell lines by NE (10 µM) and/or COR (10 µM) treatment for 48 h *p < 0.05, **p < 0.01.

To gain further insight into the relationship between depression and cancer, especially the genes in the cancer-related signaling pathways, we used the most important stress-related hormones, norepinephrine (NE) and cortisol (COR) to mimic the effect of chronic stress in various cancer cells. Results showed that NTRK1 (**Figure 7A**), an important signaling molecule involved in many signaling pathways was downregulated, while S100A9 was upregulated by COR or NE treatment in MCF-7, MDA-MB-231, A549, and SW480 cell line (**Figure 7B**). Similar results were found in SFRP5, SLC45A3, and FRZB (**Figures 7C–E**). All these results were consistent with their expression in cancer tissues in TCGA (**Supplementary Figures S1A–F**). CircRNA hsa_circ_0087100 derived from CNTNAP3, is important in synapse development and social behaviors, was induced by COR or NE treatment (**Figure 7F**). circRNAs hsa_circ_0014221 and hsa_circ_0014220 both derived from S100A9, involved in the IL-7 signaling pathway were also increased by COR or NE inducing (**Figures 7G, H**).

**Figure 8:**
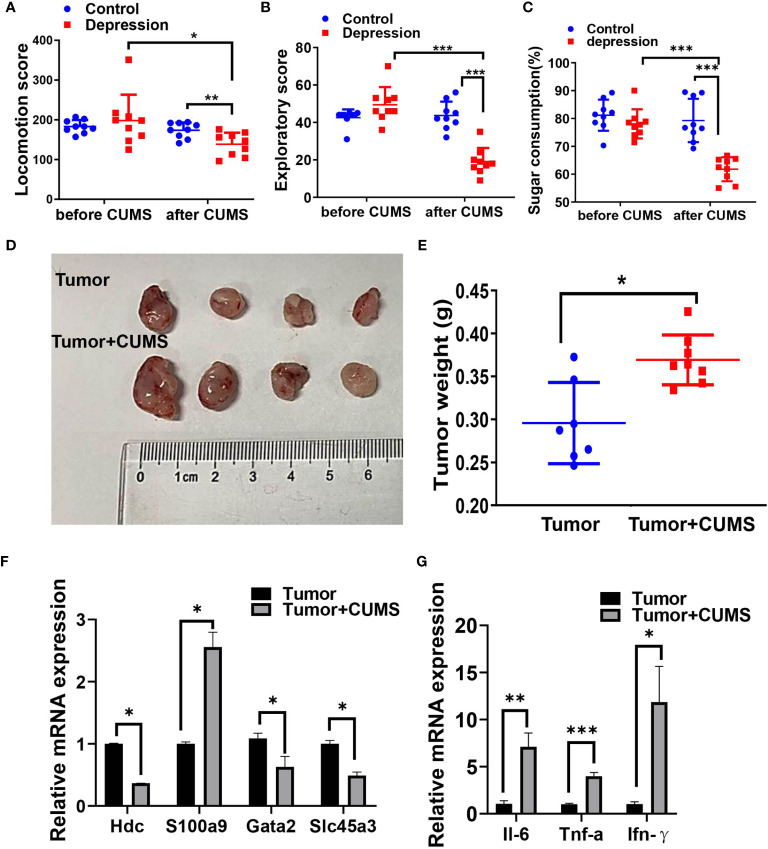
CUMS promotes tumor growth in vivo. Locomotion score **(A)** and exploratory score **(B)** of mice expose (or not) to CUMS. **(C)** Sugar consumption of mice in two groups exposed (or not) to CUMS. **(D, E)** The tumor-growth promotion effect of CUMS was compared between tumor and tumor + CUMS mice engrafted with LLC (n = 9 for each group). **(F)** Cancer-associated gene levels between the two groups by qRT-PCR. **(G)** Proinflammatory factor levels between the two groups by qRT-PCR. *p < 0.05, **p < 0.01, ***p < 0.001.

Next, we conducted CUMS plus tumor-bearing mouse model to investigate the impact of depression symptoms on outcomes of cancer. As it is shown in **Figures 8A–E**, mice exposed to CUMS revealed a declining of exercise capacity and sugar consumption as well as an enlargement of the tumor weight. Cancer-related genes HDC, GATA2, and SLC45A3 were downregulated and S100A9 was upregulated by CUMS **(** **Figure 8F**). Expression levels of proinflammatory factors IL-6, IFN-γ, and TNF-α were also increased by CUMS compared with tumor group **(** **Figure 8G**).

### Validation of Cancer-Associated Genes in the ceRNA Network in Cancer Cell Lines

**Figure 9:**
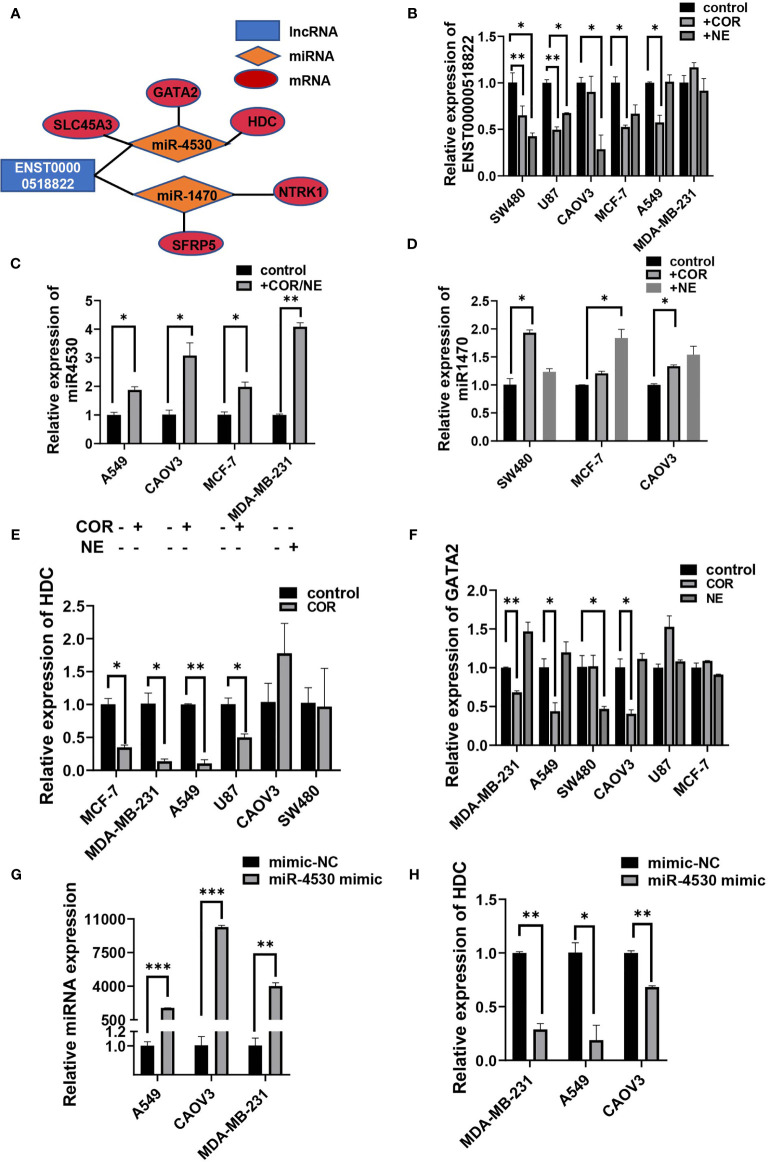
Validation on regulatory relationship of cancer-associated ceRNA networks. **(A)** ceRNA network of some cancer-related genes. qRT-PCR analysis of the relative expression of ENST00000518822 **(B)** miR-4530 **(C)** and miR-1470 **(D)**, HDC **(E)**, and GATA2 **(F)**. miR-4530 **(G)** and HDC **(H)** in A549, CAOV3, and MDA-MB-231 cells by miR-4530 mimics. *p < 0.05, **p < 0.01, ***p < 0.001.

Some of the tumor-associated genes were in the ceRNA network. Specifically, four lncRNAs, seven miRNAs, and 19 mRNAs that are tumor-related genes were included in the ceRNA networks (**Supplementary Table S9**). Our observations predicted that lncRNA ENST00000518822 acts as a sponge for miR-4530, so as to regulate HDC, GATA2, and SLC45A3 expression. ENST00000518822 may also regulate NTRK1 and SFRP5 by binding to miR-1470 (**Figure 9A**). So, we further investigated the above gene expression in several cancer cell lines under the inducing of COR or NE. As expected, lncRNA ENST00000518822 was significantly restrained by COR or NE treatment in MCF-7, A549, SW480, U87, and CAOV3 cell lines (**Figure 9B**). Conversely, miR-1470 and miR-4530 were induced by COR or NE (**Figures 9C, D**). While HDC and GATA2 were significantly attenuated by COR or NE (**Figures 9E, F**). To verify the relationship between miRNA and its target RNA, we performed miR-4530 mimics in three different cancer cell lines (**Figure 9G**). We found that miR-4530 overexpression significantly reduced HDC expression in A549, CAOV3, and MAD-MB-231 cells (**Figure 9H**).

## Discussion

The pathogenesis of depression is complicated, which comprised a combination of genetic, psychological, environmental, biological, and cultural factors. The recognized mechanism of depression involves monoamine neurotransmitters (serotonin, noradrenaline, and dopamine) (17) and HPA axis hypothesis (plasma cortisol) (18). Our results found that noradrenalin and 5-HT levels were lower while cortisol was higher in depression patients than in healthy ones which is consistent with the recognized mechanism of depression. Additionally, the view of inflammation driving the development of depression has been widely accepted. Stress-induced releasing of catecholamines and glucocorticoid resistance result in inflammasome activation, so as to reduced synaptic availability of the neurotransmitters (19). A variety of proinflammatory cytokines such as IL-1β, IL-6, TNF, and Toll-like receptor 3 (TLR3) has been found to overexpress depression (20). Therefore, there have been calls for peripheral blood IL-1β, IL-6, and TNF as biomarkers of depression patients (21). In this study, results for IL-6, IFN-γ, and TNF-α in plasma were also compatible with the inflammation theory of chronic stress-induced depression.

In recent years, numerous studies have confirmed that circRNAs, lncRNAs, and miRNAs play crucial regulatory functions in the initiation and development of depression. For example, has_circRNA_103636 is recognized as a valuable biomarker of depression and potential target for the exploration of antidepressants (22). Neuronal-enriched gene LINC00473 was found to be downregulated in the prefrontal cortex of depressed females (23). miR-1202 was reported to be downregulated in the brain of depression patients which suggests it as a potential target for novel antidepressant treatments (24). Herein, we examined the complexity of the transcriptome, including circRNAs, lncRNAs, miRNAs, and mRNAs of depression and healthy individuals using gene chip technology. We identified 340 circRNAs, 398 lncRNAs, 206 miRNAs, and 92 mRNAs that were differentially expressed between the two groups. Some of these transcripts are closely related to behavioral and cognitive dysfunction. The contactin-associated protein-like (CNTNAP) family members CNTNAP2 and CNTNAP3 were among the DE mRNAs. It is well known that the CNTNAP family is implicated in mood disorder. CNTNAP3 plays a key role in synapse development and social behaviors (25), while knockdown of CNTNAP2 resulted in decreasing of neurite length (26). Both the two genes and has_circ-0087100 (hostgene: CNTNAP3) were dysregulated in the depression group. GO analysis indicated that the DE RNAs are involved in positive regulation of neuron differentiation, neurongenesis, neutrophil degranulation, and nervous system development. KEGG enrichment revealed that the important signaling pathways such as hippo, MAPK, Wnt, PI3K/Akt, cGMP-PKG, RAS, and IL-17. All of these pathways are involved in the regulation of hippocampal function or pathological processes associated with depression (27–32), indicating the pivotal roles of DE RNAs in depression pathogenesis. Further studies are needed to demonstrate the intricate functions of these DE RNAs.

Plenty of works have suggested that depression is related to worse outcomes in cancer patients, so we paid much attention to the DEGs associated with cancer. Our results showed that some tumor-associated genes were dysregulated in depression patients. For example, S100A9 acts as a potent amplifier of inflammation in cancer progression as well as tumor spread (33) and has been reported to strongly upregulate in many tumors. In this study, S100A9 was not only upregulated in the depression group but also increased in cancer cell lines with NE/COR treatment as well as in tumor-bearing mice with chronic stress, which was consistent with previous researches (34–36). Additionally, the relative expression of S100A9 was positively correlated with HAMD score of the depression samples, which may suggest a positive correlation between depression and cancer. Moreover, has_circ_0014220, has_circ_0014221, and their host gene S100A9 enriched in the IL-17 signaling pathway, augmented levels of the inflammatory cytokines IL-6, TNF-α, and IFN-γ in depression patients as well as tumor-bearing mice with CUMS. All these results support the inflammatory theories of depression accelerating cancer progression (6).

It is noteworthy that plenty of cancer-related pathways were the most important pathways in the KEGG pathway analysis of DEGs. KEGG analysis of DE circRNAs shows enrichment in prostate cancer, colorectal cancer, and bladder cancer; KEGG analysis of DE lncRNAs shows enrichment in small cell lung cancer and prostate cancer; KEGG analysis of DE miRNAs shows enrichment in breast cancer; and KEGG analysis of DE mRNAs shows enrichment in thyroid cancer. Also, many tumor-associated pathways were enriched such as the Wnt, MAPK, and PI3K-AKT signaling pathways, EGFR tyrosine kinase inhibitor resistance, etc. GO and KEGG pathway analyses of the DEGs in ceRNA network also involved in several pathways associated with cancer, such as RAS, Wnt, MAPK, and PI3K-AKT signaling pathways. Some of these pathways are playing important roles in both depression and cancer, such as RAS signaling pathway (31, 37) and IL-17 signaling pathway (32, 38). So, we performed stress-induced cancer progression *in vivo* and *in vitro* experiments to investigate the mRNA expression involved in the pathways. The results of NTRK1 level in cancer cell lines demonstrated that stress hormones inhibited NTRK1 expression. NTRK1 is a key gene in many signaling pathways such as MAPK, PI3K-AKT, and RAS. SFRP5 belonging to the Wnt signaling pathway was found to be declined by stress hormone treatment (39). It is interesting that results of cancer-associated genes expression in different cell lines showed a more pronounced COR treatment compared with NE treatment, indicating that maybe glucocorticoids play a more prominent role than the adrenergic system, which was consistent with a previous report (40).

Some cancer-related circRNAs and lncRNAs previously reported were also identified by our research. For instance, has_circ_0007334 (circMBOAT2) was reported to play an oncogenic role in prostate cancer and colorectal cancer (41, 42). We observed that has_circ_0007334 was highly expressed in the depression group, suggesting its potential role in depression-promoting cancer. LncRNA LINC00963 has been considered an oncogene in many cancers and was upregulated in the depression group in our research (43–45). As we have known, circRNAs and lncRNAs can compete with miRNA through MRE to form a ceRNA network and play a regulatory function in various diseases (46). We constructed ceRNA of circRNA/lncRNA-miRNA-mRNA networks and found that many oncogenes and tumor suppressors participate in the ceRNA network. Overall, four lncRNAs, six miRNAs, and 18 mRNAs that are tumor-related genes were included in the ceRNA networks. We preliminary explored one of the networks by overexpression of miR-4530, to discover that HDC was downregulated in three cancer cell lines. HDC was enriched in histidine metabolism. Histidine decarboxylase (HDC)-deficient CD8 T cell suppresses proliferation and induces prostaglandin E2 (PGE2) expression level in glioma (47). Insufficiency of HDC inhibits myeloid cell maturation as a result of promoting tumor growth (48). Further study is needed to uncover the mechanism of miR-4530 in cancer progression by targeting HDC.

ncRNAs play important roles in the pathogenesis of many diseases including depression and cancer, which may mark these ncRNAs as potential target of therapeutic drugs for depression-related cancer comorbidity. Nonetheless, this research has some limitations such as the lack of human samples of depression comorbidity with cancer and the need for in-depth study of the ncRNAs; thus, future study will address the mechanism by which ncRNAs promote tumor progression in depression comorbidity tumor, focusing specifically on ceRNA networks.

In summary, our study presented the aberrant expression profiles of circRNAs, lncRNAs, miRNAs, and mRNAs in depression compared with healthy ones. Numerous specific DEGs were associated with cancer and were validated in stress-induced cancer cell lines as well as tumor-bearing mice with CUMS. All these findings may elucidate the mechanism of depression which worsens cancer from a novel perspective: ncRNAs and ceRNA hypothesis. The specific DEGs could act as biomarkers and potential therapeutic targets of depression in cancer comorbidity for future studies through the pathways of IL-17 or histidine metabolism.

## Data Availability Statement

The datasets presented in this study can be found in online repositories. The name of the repository and accession number can be found below: National Center for Biotechnology Information (NCBI) Gene Expression Omnibus (GEO), https://www.ncbi.nlm.nih.gov/geo/, GSE182195.

## Ethics Statement

The studies involving human participants were reviewed and approved by the Ethics Committee of Fengxian Hospital, Southern Medical University. The patients/participants provided their written informed consent to participate in this study. The animal study was reviewed and approved by Institutional Ethics Committee of Fengxian Hospital, Southern Medical University.

## Author Contributions

FX and CH designed the study. TZ, ML, JC, and WZ collected patient samples and performed depression scales. ZY, ZG, YC, WC, and PH conducted the animal model, performed RNA extraction, qRT-PCR, and ELISA assay. TZ, ZX, and JD performed data analysis. TZ wrote the manuscript. TZ and FX supervised the project. All authors contributed to the article and approved the submitted version.

## Funding

This work was supported by the Shanghai Municipal Health Commission (No. 20194Y0446), Fengxian District Science Commission (No. 20201607), and Shanghai Municipal Science Commission (No. 19411971700).

## Conflict of Interest

The authors declare that the research was conducted in the absence of any commercial or financial relationships that could be construed as a potential conflict of interest.

## Publisher’s Note

All claims expressed in this article are solely those of the authors and do not necessarily represent those of their affiliated organizations, or those of the publisher, the editors and the reviewers. Any product that may be evaluated in this article, or claim that may be made by its manufacturer, is not guaranteed or endorsed by the publisher.

## Supplementary Material

Supplementary Figure 1 HDC, S100A9, SFRP5, SLC45A3, NTRK1, and GATA2 mRNA expression in various cancer tissue in TCGA. **(A)** OV, **(B)** BRCA, **(C)** COAD, **(D)** LUAD, **(E)** BGMLGG, and **(F)** LUSD.
