# Supplementary figures and images for: Chronic Stress-Induced Gene Changes In Vitro and In Vivo: Potential Biomarkers Associated With Depression and Cancer Based on circRNA- and lncRNA-Associated ceRNA Networks
Source: Front Oncol. 2021 Sep 28;11:744251. doi: 10.3389/fonc.2021.744251 (PMC8507324; doi:10.3389/fonc.2021.744251)

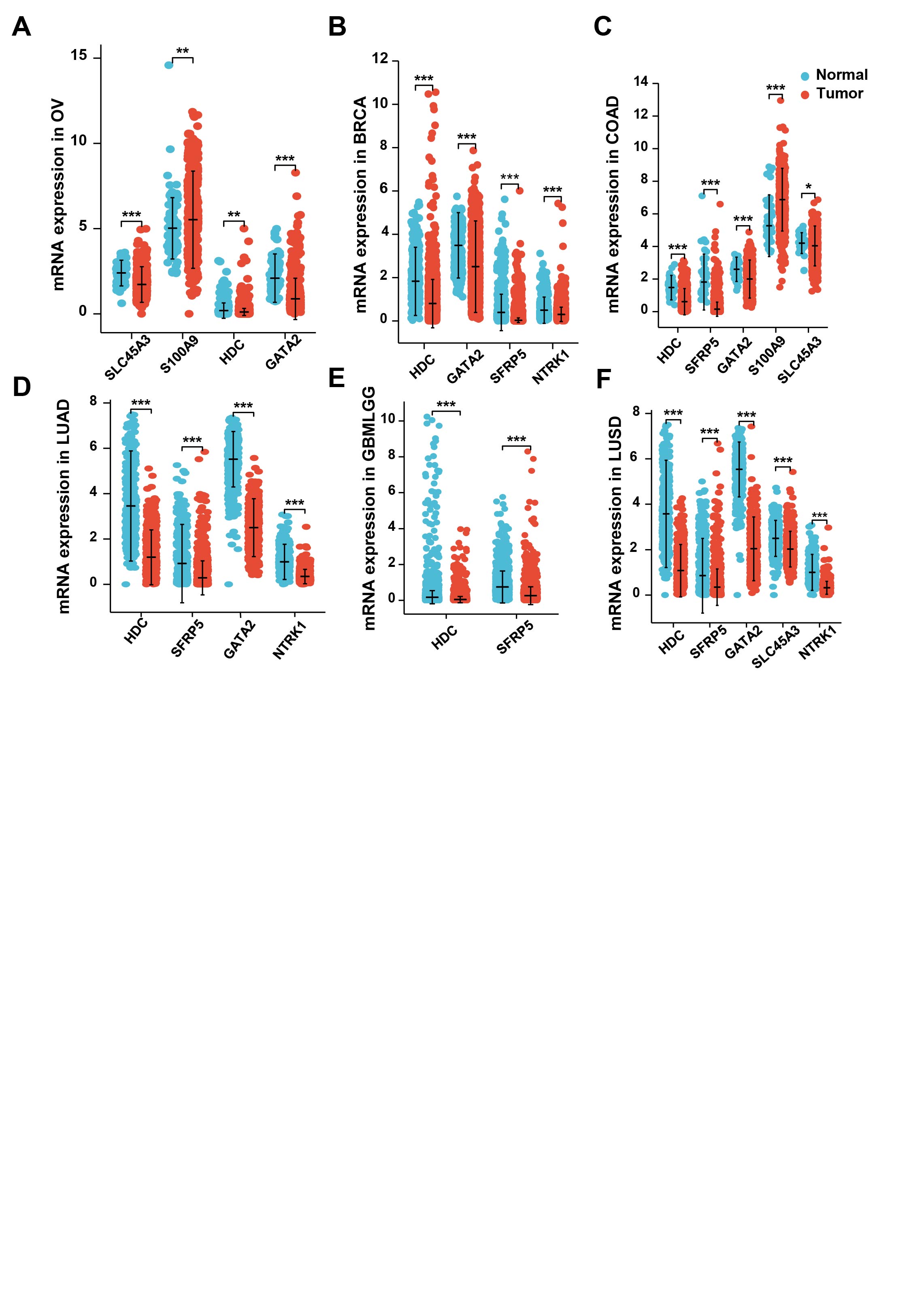

Supplement: Supplementary Figure 1 — HDC, S100A9, SFRP5, SLC45A3, NTRK1, and GATA2 mRNA expression in various cancer tissue in TCGA. (A) OV, (B) BRCA, (C) COAD, (D) LUAD, (E) BGMLGG, and (F) LUSD. [file Image_1.jpeg]
