# Supplementary material for: Chronic Stress-Induced Gene Changes In Vitro and In Vivo: Potential Biomarkers Associated With Depression and Cancer Based on circRNA- and lncRNA-Associated ceRNA Networks
Source: Front Oncol. 2021 Sep 28;11:744251. doi: 10.3389/fonc.2021.744251 (PMC8507324; doi:10.3389/fonc.2021.744251)
Supplement: Supplementary file 3 [file Table_1.docx]

Supplementary TableS1 Primers of the RNAs

| RNA |  | primer |
| --- | --- | --- |
| HDC | F | TACTGGGTTCTGGGTCAAGG |
|  | R | GAATTTCAAAGGAAGGGTCGT |
| S100A9 | F | TCATCAACACCTTCCACCAAT |
|  | R | TCACCCTCGTGCATCTTCTC |
| GATA2 | F | TCCTGACCCTAGCACCACG |
|  | R | GGGACTGCCACTTTCCATCT |
| NTRK1 | F | TCCAACACGGAGGCAATC |
|  | R | AGCCCAGGACATCCAGGTAG |
| FRZB | F | AGATGCCCAACCACCTGC |
|  | R | CACGCCCCTGTCGTACACT |
| KRT1 | F | ATTTGCCTCCTTCATTGACAAG |
|  | R | GTTCCGAATCCAACCGAGA |
| hsa-miR-595 | F | AGTGTGCCGTGGTGTGTCTAA |
|  | R | TTTTTTTAGACACACCACGGC |
| hsa-miR-575 | F | GAGCCAGGGACAGGAGCA |
|  | R | TTTTTTTGCTCCTGTCCCTG |
| hsa-miR-1238-5p | F | GTGAGTGGGAGCCCCAGTG |
|  | R | ACTGGGGCTCCCACTCAC |
| hsa-miR-718 | F | CTTCCGCCCCGCCG |
|  | R | TTTTTTTTTTTTTCGACGCC |
| hsa-miR-4534 | F | GGATGGAGGAGGGGTCTAAA |
|  | R | TTTAGACCCCTCCTCCATCC |
| hsa-miR-3937 | F | ACAGGCGGCTGTAGCAATG |
|  | R | CATTGCTACAGCCGCCTGT |
| hsa_circ_0014220 | F | GCTGGGGCCAAATAAAGTCT |
|  | R | GTTCCAGCTGCGACATTTTG |
| hsa_circ_0071253 | F | GATCTTACCCGTGACAAAATGTG |
|  | R | TTGGTTCCTTGGGTCCTGG |
| hsa_circ_0069475 | F | CCACCTTGGGCATGTCATCT |
|  | R | CCACACAACGCTTGGAACG |
| hsa_circ_0026463 | F | AGGTCTTAAACTCCCCATTTCCT |
|  | R | GCTGCCACCTCCACTGAT |
| hsa_circ_0029644 | F | GGCAGAAGACCTCCAGTACA |
|  | R | TTCATCTGTCTCTGGAGCCA |
| hsa_circ_0025413 | F | AAGTCAGCAGCAACCATGTC |
|  | R | GCATAGATGAGCAACCGAGC |
| hsa_circ_0008847 | F | GGAGTGGAGAACATGCACAA |
|  | R | AAGGCAAAGAGTTGGCACAC |
| NR_003672 | F | CCAGGTAAGAGGTGAGGCGGAGG |
|  | R | CCACAGGCACAGCAGAGCAACT |
| lnc-SETD4-11 | F | GCTGCCACAGAACCCATAGTA |
|  | R | ACGCTTGAACACGCTGAATA |
| LINC00963 | F | TTTAGTAGAGACGGGGTTTCACT |
|  | R | TGGATCACAAGGTCAGGGG |
| RPPH1 | F | ACTCCACTCCCATGTCCCTT |
|  | R | TGCCCAGTCTGACCTCGC |
| lnc-GRIA3-2:1 | F | CAGATCCTCAATCAAGCCAAGA |
|  | R | CATACAGTGCTGCTCCAGTACCT |
| Actin | F | AGCCTTCCTTCCTGGGTATG |
|  | R | GAGGTCTTTACGGATGTCAACG |
| U6 | F | CTCGCTTCGGCAGCACA |
|  | R | AACGCTTCACGAATTTGCGT |
| SFRP5 | F | CCAGTGACTTTGTGGTCAAAAT |
|  | R | CTTCTTTTTCTGGGCTCCAATC |
| SLC45A3 | F | CATGACCTTCACGCTGTTTTAC |
|  | R | AACGCCTTCATCATAGTGTCTC |
| has_circ_0007334 | F | GGAGTGGAGAACATGCACAA |
|  | R | AAGGCAAAGAGTTGGCACAC |
| has_circ_0014221 | F | CCTGCCTCTACCCAACCAGG |
|  | R | CTCCTCGAAGCTCAGCTGCT |
| has_circ_0014220 | F | TGTCTTGGCTGTGGGGCTAG |
|  | R | TGGTTCAGGGTGTCTGGGTG |
| has_circ_0087100 | F | TGGGCACGTTCGTTTACAGC |
|  | R | GCATCCCAATCCTGCCCCTA |
| ENST00000518822 | F | ATCGATACAGAGCATCTGCACTG |
|  | R | GGTCCAAGGCCGACTATACC |
| Il-6 (mouse) | F | CAGTTGCCTTCTTGGG |
|  | R | AGACAGGTCTGTTGGGAG |
| Tnf-a (mouse) | F | TAACTTAGAAAGGGGATTATGGCT |
|  | R | T GGAAAGGTCTGAAGGTAGGAA |
| Ifn-γ (mouse) | F | GACATGAAAATCCTGCAGAGC |
|  | R | TGAGCTCATTGAATGCTTGG |
| Hdc (mouse) | F | TTAAGCTGTGGTTTGTGATTCG |
|  | R | CTGTGAGACAATTAGGACCCTT |
| S100a9 (mouse) | F | CACAGTTGGCAACCTTTATGAA |
|  | R | TCATACACTCCTCAAAGCTCAG |
| Gata2 (mouse) | F | CACAAGATGAATGGACAGAACC |
|  | R | ACATTGTGCAGCTTGTAGTAGA |
| Slc45a3 (mouse) | F | GTTCCTGTTGTCCTGTGTCCTGTG |
|  | R | TCGTTCTGTAAGTCCTGCGTTCTTC |

Supplementary Table S2**.** Demographic and clinical characteristics of mental healthy volunteers and depression patients.

|  | healthy volunteers (n=27) | | Depression patients (n=26) | |
| --- | --- | --- | --- | --- |
|  |  |  |  |  |
| Age | 47.10±10.38 | | 52.32±12.90 | |
| Sex (n(%)) | |  |  |  |
| -Females | 13(48.15%) | | | 11(42.31%) |
| -Males | 14(51.85%) | | | 15(57.69%) |
| Length of disease  (years, means) |  | | | 8.70±6.91 |
| HAMD24 score | 0.45±0.99 | | | 41.41±11.34** |
| Total Life Events score | 24.56±10.21 | | | 33.34±12.77* |
| Negative Life Events score | 15.87±9.98 | | | 36.79±10.26** |

*P<0.05, **p<0.01 compared to mental healthy volunteers.
